# Health and Retirement in Europe

**DOI:** 10.3390/ijerph6102676

**Published:** 2009-10-20

**Authors:** Ronald Hagan, Andrew M. Jones, Nigel Rice

**Affiliations:** 1 School of Women’s and Infants’ Health, University of Western Australia, Perth, WA 6008, Western Australia; E-Mail: ronnie.hagan@gmail.com; 2 Department of Economics and Related Studies, University of York, YO10 5DD, UK; 3 Centre for Health Economics, University of York, YO10 5DD, UK; E-Mail: nr5@york.ac.uk

**Keywords:** retirement, health, ECHP, discrete time hazard models

## Abstract

We use discrete-time hazard models with internationally comparable data from the full eight waves of the European Community Household Panel (ECHP) to study the relationship between retirement and health in nine European countries. Our results provide new evidence of the relationship of health shocks to early retirement. The pattern of results across countries reflects international differences in the incentives created by social security systems.

## 1. Introduction

The developed world is facing an ageing population as life expectancy increases and birth rates decrease. At the same time, the average age of retirement has been decreasing in Europe and recent estimates range from 58 in Luxembourg to 63 in Sweden [1]. This has important implications for the labour market and will increase demands on both the health system and the public provision of pensions and other benefits [2,3]. Countries differ in the financial incentives provided by their public pension systems, in the nature of disability and incapacity benefits, and in how the system drives early retirement [4–8] There are differences in life expectancy, activity rates for older workers, retirement age, employment protection legislation, macroeconomic conditions and projected dependency rates between European countries [8]. Within the European Union (EU) the old-age dependency rate is forecast to double between 2000 and 2040 [9]. These pressures vary between member states but are becoming a major political issue in all countries.

Ill-health is frequently cited as a cause for retirement [3,10]. Many studies have explored the relationship between level of health and retirement, with the earlier work summarised in [11] and more recent work summarised by [12,13]. The relationship between the measure of health used and ‘true’ health has been a constant concern for researchers. The commonest measure of health used in early studies was the subjective measure of self -assessed health (SAH) which is asked both in general terms and in relation solely to the ability to perform work activities. This raises potential problems of both validity and reverse causality. Those who are inactive may have an incentive to report worse than actual health to justify their inactivity (known as ‘justification bias’). Health itself may be effected by the labour market status of the individual, depending on the degree of self-esteem generated by the job or the amount of stress or occupational risk associated with the job or work environment.

However the literature is conflicting on the extent of state-dependent reporting bias in subjective measures of health [2,10,14–17]. Faced with this uncertainty, measures of health that are believed to be more objective have been proposed and used. These have included self-reports on specific medical conditions and functional limitations, or the use of various symptom checklists. Again, the published evidence is conflicting [18,19] and these measures have also been shown to have some measurement bias [20].

More recent studies have constructed an underlying ‘health stock’ for each individual and tracked temporal changes in this measure as a proxy for individual ‘health shocks’ that might influence retirement behaviour [21–24]. These latent measures of health are created using predicted values from estimated models of SAH, using as predictors health indicators that are related to reports of specific medical conditions and functional limitations. Demographic variables, but not employment status, have also been used with the belief that this removes the effect of employment status on reporting behaviour. Hence the resulting predictions are claimed to be free of any justification bias. This however will only be true if the health indicators and demographic variables are themselves not directly related to the measurement error. These recent studies have looked at the dynamics of the health and retirement relationship focusing on the relative contributions of long-run health and of acute changes in an individual’s health stock. Using longitudinal data from individual countries including the USA, UK and Canada they show that the dynamics of health are important and that changes in health play an important role in retirement decisions. These studies have underlined the necessity of having reliable panel data available to model the relationship between health and employment status.

A range of alternative outcome measures have been used to define retirement. The most direct is self-reported retirement, though this may be vulnerable to recall bias and may not accurately define observed prolonged work inactivity [25]. More commonly, labour force participation has been assessed in terms of transitions from being active in the labour market to various forms of inactivity. These have included early retirement programmes, disability insurance programmes or unemployment programmes [2,21]. Additional outcome measures have included the expected age of retirement [15] and the expected probability of being in full-time work at age 62 years [16]. Health may have different effects depending on the outcome variable chosen [2,21] and how other covariates behave may depend on the outcome and health measures used. This has been commented on in the literature but with conflicting results [2,22].

A basic requirement for studies on the impact of health on retirement decisions is the availability of good data sources. The USA has had a long tradition of gathering data specifically on older members of the community, with European countries only recently establishing similar datasets with the Survey of Health, Ageing and Retirement in Europe (SHARE) and the English Longitudinal Study of Ageing (ELSA). Data incompatibility problems have resulted in almost all published studies relating to data from an individual country hence making comparisons between countries difficult. Some preliminary results have been presented using two European datasets with work from [26] on the first wave of the SHARE data and from [27] on comparisons of retirement decisions of older European couples using one wave of the European Community Household Panel (ECHP). However it will be some years before the ELSA and SHARE data provides sufficient longitudinal variation to match the eight years available of the ECHP.

At an analytical level, recent applications of discrete-time proportional hazard models have provided a flexible dynamic structure within which to examine these relationships in appropriately structured panel data. This approach has been used, with ‘stock samples’ of individuals within a target age range, with data from the British Household Panel Survey [23,24].

In this paper we use discrete-time mixed proportional hazard models with internationally comparable longitudinal data from the full eight waves of the European Community Household Panel (ECHP) to study the relationship between retirement, health stock and health shocks in nine European countries. The stock sample approach conditions on individuals within a set age range and who are active in the labour market at the first wave of data. A variety of health measures are used, as are alternative definitions of retirement. The availability of panel data allows us to exploit the timing of events in order to identify the impact of health shocks on retirement [28]. Self-reported health shocks are recorded prior to the individual‘s date of exit from the labour market and this should help to mitigate the influence of justification bias, although changes in reporting due to anticipation of future retirement may still be a source of bias.

Our work provides comparisons of the effects of health across a range of European countries based on comparable panel data. It provides further evidence on and confirms the relationship between health and health shocks and the retirement decision. The results show that the effects of other covariates in the models, especially financial factors, are robust across specifications using different measures of health and health shocks. We demonstrate the effect of using alternative definitions of retirement as the outcome measure when assessing the effects of individual factors. There is consistency in the effects of the health measures across the two definitions of retirement and in the effects of the other factors including financial factors and education. Health shocks have a smaller impact on the hazard of retirement in the countries that have the strongest financial incentive to retire early, although it is notable that the countries where the impact of health shocks on the hazard of retirement is highest (Ireland, Portugal) have high ratios of disability-related to total pensions.

## 2. ECHP Data

Data drawn from the full eight waves (1994–2001) of the European Community Household Panel (ECHP) dataset are used. The ECHP is a standardized annual longitudinal survey carried out among the pre-enlargement member countries of the European Union (EC-15). The ECHP was designed and coordinated by the Statistical Office of the European Communities (Eurostat) covering, at the level of households and individuals, information on demographics, participation in the labour market, income, health, education and training, housing etc. With some minor exceptions, the same sampling methodology and an identical questionnaire was used in each participating member state [29–31]. The first wave was conducted in 1994 and the User’s Data Base (ECHP-UDB), which is an anonymised and user-friendly version of the data, has been available since December 1998. The version containing the complete eight waves (December 2003) is used in this study. Nine countries were selected for study, listed in alphabetical order: Belgium, Denmark, France, Greece, Ireland, Italy, Portugal, Spain and the UK. These particular countries were selected as they all had data from the first wave of the panel in relation to health variables and employment activity. For the UK we use the comparable sample of BHPS data that is supplied with the ECHP-UDB, rather than the original ECHP sample which is only available for 3 waves.

### 2.1. Selection and Creation of Variables

#### 2.1.1. Health variables

The ECHP has a variety of health-related questions. These include a measure of general self-assessed health (SAH) status as well as more specific questions related to limitations in daily activities, the presence of recent illness due to physical, emotional or mental health problems as well as admission to hospital.

The SAH variable is a simple five-point scale based on answers to the question “How is your health in general?” The available answers were very good/ good/ fair/ bad/ very bad. The question wording in France was “Pourriez vous indiquer, sur une échelle allant de 1 (pas satisfait du tout) à 6 (très stisfait) votre degree de satisfaction en ce qui concerne les points suivants? …… Votre santé” the answer was on a 6 point scale and has been recoded in the UDB to a five point scale. This variable was recoded to be increasing in good health.

The remaining health questions predominantly focus on impairment or limitation in normal daily activities. These consist of a set of self-report questions firstly based on the question “are you hampered in your daily activities by any physical or mental problem, illness or disability? coded: 1 yes, severely; 2 yes, to some extent; 3 no; −9 missing. In France the question was worded “gene par une maladie chronique, un handicap?” Binary variables for each of the three possible answers had been created i.e., severely hampered for answer =1; moderately hampered for =2; not hampered for =3. The next two questions were yes/no answers related to recent health problems and again binary dummy variables had been created. The questions were worded “during the past two weeks, have you had to cut down things you usually do about the house, at work or in free time because of illness or injury?” and “during the past two weeks, have you had to cut down things you usually do about the house, at work or in free time because of an emotional or mental health problem?”. The final health indicator, also coded as a binary variable, was worded “during the past 12 months, have you been admitted to a hospital as an in-patient?”.

It was emphasised earlier that using measures of self-reported health that are recorded prior to an individual’s exit from the labour market should help to mitigate problems of justification bias. However there may be anticipation effects and measurement errors may still occur. To address this possibility the method of estimating a model of SAH as a function of a set of health indicators is used to define a latent ‘health stock’. This follows the approach of [32] as implemented in [21] and more recently used by [23] and [24]. There are differences in how the latter two studies have created their latent health variable. [23] use personal characteristics as well as health indicators while [24] only include the health indicators when constructing the health stock. In this study we adopt the approach of [23]. The idea of constructing this health stock variable is analogous to using the health indicators as instrumental variables to purge measurement error in the SAH variable. More conservatively it can be seen as a way of reducing the dimensionality of the problem by forming a single linear combination from a set of health indicators.

To construct each individual’s latent health stock we consider the aspect of health that influences an individual’s decision to retire, H^R^_it_, to be a function of the health indicators, Z_it_, such that:

(1) $$\begin{array}{cc} {\text{H}}_{\text{it}}^{\text{R}}={\text{Z}}_{\text{it}}^{‘}\;\beta +{ɛ}_{\text{it}}, & \text{i}=1.2,\ldots \ldots ,\;\text{n};\;\text{t}=1,2,\ldots \ldots ,\;{\text{T}}_{\text{i}} \end{array}$$

where ɛ_it_ is a time varying random error term that is assumed to be uncorrelated with Z_it_. H^R^_it_ can not be directly observed but instead we have a measure of self-assessed health (SAH), H^S^_it_ and we can specify the latent counterpart of this as H^*^_it_ such that:

(2) $${\text{H}}_{\text{it}}^{*}={\text{H}}_{\;\text{it}}^{\text{R}}+\eta_{\text{it}}$$

where the random error term η_it_ represents measurement error in the mapping of H^*^_it_ to H^R^_it_ and is uncorrelated with H^R^_it_. Substituting (1) into (2) gives:

(3) $${\text{H}}_{\text{it}}^{*}={\text{Z}}_{\text{it}}^{‘}\;\beta +{ɛ}_{\text{it}}+\eta_{\text{it}}={\text{Z}}_{\text{it}}^{‘}\;\beta +\nu_{\text{it}}$$

The presence of η_it_ in (3) is a potential source of bias if H^*^_it_ is used directly when estimating the impact of health on retirement. This can either be distributed independently of labour market status or be a function of the labour market status of the individual. If it were distributed independently it would represent classical measurement error and could attenuate the effect of health on retirement. Alternatively, if it is a function of the labour market status i.e., individuals rationalise early labour market exit by reporting ill-health, then this would overestimate the effect of health on retirement. To avoid this bias a predicted health stock is used.

Combining (3) with the observation mechanism linking the categorical indicator, H^s^_it_, to the latent measure of health H^*^_it_, and assuming a distributional form for ν_it_ we can estimate the coefficients, β. For example, in the case of the categorical self-assessed health measure the observation mechanism can be expressed as:

(4) $${\text{H}}_{\text{it}}^{\text{s}}=\text{j}\;\text{if}\;\mu_{\text{j}-1}\;<\;{\text{H}}_{\text{it}}^{*}\leq \mu_{\text{j}},\;\text{j}=1,\ldots ,\text{m}$$

where μ_0_ = −∞, μ_j_ ≤ μ_j+1_, μ_m_ = ∞. Assuming ν_it_ is normally distributed, model (3) can be estimated as an ordered probit using maximum likelihood. The predicted values for the health stock can then be used in our retirement model.

The ECHP does not have the same extensive range of objective health variables as the BHPS, as used by [23] and [24], but does have a small set of measures relating to limitation in daily activities, recent illness or mental problem and a history of in-patient stay in hospital. The latent health stock measure was thus created using the health indicators and demographic variables, using the method of [23]. They estimated the health stock for each wave of the data using the wave specific values of the objective health variables but also included some demographic variables. These estimated values are then normalised as a deviation of the individual’s health index from the cohort mean for each year. This predicted individual ‘health stock’ thus creates a health stock for each individual in relation to the year on year average for the sample. The normalised variable has a mean of 0 and a standard deviation of 1 for each wave of the sample. This normalisation is carried out separately for males and females in each county in order to address concerns about cross-country and gender differences in self-reporting of health. This process was performed on the full sample.

Table 1 shows the coefficients of the health variables for the ordered probit models estimated on the first wave for both sexes in all countries for the health stock. The presence of mental health problems is the only adverse health event variable which is inconsistent in its effect. However, in those models were it is statistically significant it always has a negative coefficient. The remaining factors all have the expected negative coefficient and demonstrate variability between the sexes and between countries. The cut-points from the ordered probit models were then used to create an adjusted derived SAH for each individual in each wave.

Having the health stock variable allows us to specify different dynamic models for the impact of health on the hazard of early retirement. Our main results are based on three specifications for the health variable:

- current level of health stock
- lagged health stock
- a discrete health shock

The first specification includes the current level of the health stock variable. The second uses lagged health stock with the main results presenting models with a one-year lag and the robustness analysis experimenting with additional lags. The third specification uses the health stock variable to construct measures of discrete ‘health shocks’, reflecting acute deteriorations in health. All specifications also condition on the initial level of health stock. This allows the estimated effect of the health shocks to represent the deviation from the underlying health stock and has the advantage of helping to control for individual-specific unobserved health-related heterogeneity.

We construct measures of discrete acute health shocks by considering the differences between consecutive waves in an individual’s normalised health stock value, their reported SAH and their adjusted SAH. This is similar to the concept of an acute health shock described by [33], although she uses only reported health rather than a purged measure. Binary dummy variables were created for these shocks for each predicted health stock measure. Each is based on a decrement in the measure between consecutive waves:

- The first is based on a 1 standard deviation or greater decrement in the normalised health stock.
- The second is based on the reported (unadjusted) SAH: we compare the reported categories of SAH in waves t-1 and t and create an indicator for a reduction in SAH of 1 category or greater. This does not change between the three stock samples.
- The third is based on the adjusted SAH. The measure of health shocks for the adjusted data mimics that for the raw data. The (normalised) latent health stock is calculated for each individual at each wave. The (normalised) cut-points are then used to predict which of the 5 categories of SAH the individual is assigned to at each wave. The indicators of health shocks are then computed in the same way as step 2, but using predicted rather than actual SAH category.

The use of the second and third discrete acute health shocks means that the estimated quantitative effects of the health shocks can be compared directly for the unadjusted and adjusted measures of SAH. These binary dummy variables were then used separately in the hazard models, conditioned on the initial normalised health stock value using the final censored stock sample. Conditioning on the initial level of health stock allows us to control for heterogeneity in the retirement decision caused through individuals in worse health on entering the stock sample retiring at a greater rate than more healthy comparators. This permits us to identify better the seperate effect of a health shock.

To assess whether these measures do indeed capture acute health shocks and to ensure comparability across countries the number of adverse health events associated with each shock were counted and compared as shown in Table 2. Each acute health shock is associated with adverse health events. The health shock based on a standard deviation decrement has the highest number of adverse health events and the smallest coefficient of variation in that number across countries. This suggests that this would be the best measure in comparing countries as regards the effects of ill health on retirement decisions and this measure is used in our main results section (Section 4). However, the other two measures allow a direct comparison of the magnitude of the impact of health shocks, with and without the adjustment for reporting bias. These two measures are therefore used for robustness checks in Section 5. Comparison of the number of adverse health events associated with the unadjusted measure of SAH and the adjusted measure implies that, on average, using the unadjusted measure of decrements in SAH tends to over -report the incidence of health shocks. Not surprisingly therefore in the majority of countries there are many more health shocks recorded using the unadjusted SAH than the adjusted SAH.

#### 2.1.2. Other explanatory variables

The ECHP asked a broad range of questions related to employment. The one used in this study is based on the self-defined main activity status with individuals classifying their status as one of the following: (1) working with an employer in paid employment (15+ hours/week); (2) working with an employer in a paid apprenticeship (15+ hours/week); (3) working with an employer in training under special schemes related to employment (15+ hours/week); (4) self-employment (15+ hours/week); (5) unpaid work in a family enterprise (15+ hours/week); (6) in education or training; (7) unemployed; (8) retired; (9) doing housework, looking after children or other persons; (10) in community or military service; (11) other economically inactive; (12) working less than 15 hours. A binary variable was created based on whether the individual had selected the 8^th^ category, “retired”.

In addition, the question on the individual’s main activity status was used to generate another binary variable based on whether options 1–5, 7 or 12 were selected. This second variable uses the transition between reported activity in the labour market and inactivity as a measure of retirement: labour market activity therefore encompasses full and part-time employment, apprenticeships and training, self-employment and unemployment. This was chosen because of doubts raised about the accuracy of the self-reported ‘retired’ [25] and also because transitions from activity to inactivity have been used frequently as outcome measures in analysing the effect of health on retirement [2,21]. For both the narrow and broad definitions, retirement is recorded when the first transition occurs and the discrete time hazard model only uses observations up to that wave.

A range of income variables are used. The ECHP-UDB includes some imputation to deal with item non-response, especially for non-labour components of total income. [34] describe the imputation procedures and find that they are generally reliable in the 2003 version of the UDB. The starting point is household income from all sources which is used across all waves in which an individual is observed. This is then split into personal and other income. To permit comparisons across countries and across time the income variables are adjusted for the consumer price index (CPI) and purchasing power parities (PPPs), then equivalised by the modified-OECD scale to adjust for household size and composition. In order to reduce concerns over reverse causality we have used the one-period lagged value of these variables in all models. Other household income is used to capture the influence of the spouse’s income. In addition to income, household wealth is proxied by home ownership which is included as a binary variable.

The following socio-demographic variables are used in the analysis. Educational attainment graded using the highest grade of education achieved on the 3 level ISCED scale—completed third level secondary education; completed second stage of secondary education; completed less than second stage of secondary education—converted to binary variables; age dummy variables; the number of children living in the household; and cohabitational status as a binary variable.

#### 2.1.3. Creation of samples

The focus of this study is on the role of health in the decision to retire and thus we need to observe individuals who are active at the start of the sample and are tracked over a period when they are at a risk of retiring. This defines a stock sample [35].

For the purposes of our analyses we created three stock samples. We first selected those individuals who at wave 1 had the following characteristics: responded to the survey questionnaire; were aged 45–59 years and had measures of health and employment activity recorded for all subsequent waves. This age group was perceived to be at risk of retirement at the first wave of observation. This sample is used for the estimation of the health stock. Then, once an individual retired or was missing (lost to follow-up) their data after that point was excluded. We next selected from that stock sample those individuals who were employed or self-employed in wave 1 and finally we right censored that sample using the standard public retirement ages for each sex in each country (taken from [7]). This means that people leave the risk set when they hit the official retirement age, so that the focus of analysis is on early retirement. This defines the final estimation stock sample.

Table 3 presents summaries of the samples broken down by country and gender. The nine countries selected for study have a total of 105,613 participants in the ECHP. Of these 23,405 (11,346 males; 12,059 females) met the age and complete data requirements selection criteria with 13,766 (8,928 males; 4,850 females) meeting the additional employment criteria at wave 1. Our final estimation sample presented for analysis regarding retirement totalled 12,153 (52% of age group). This varied by country with a high of 76% in Greek and Portugese males of the eligible age group compared to the low in Irish and Spanish females of 21% and 22% respectively. The proportion of those analysed who retired during the eight-year time period varied by country and by definition of retirement with a high of 40% of Greek males self-reporting retirement compared with a low of 13% in Irish males. Just under a quarter of the sample reported retiring during the study period. However more became inactive in the labour market with 29% doing so during the study period. Overall, across the nine countries, there was a 22% increase from 2,828 to 3,464 individuals retiring depending on the definition though there was great variation between countries and between sexes.

The mean age of individuals in each country’s stock sample is similar though there are differences in the distribution across the age groups. The differences in employment status, in the reported self-assessed health status and the proportion having some degree of limitation because of health problems are similar to those reported by [36] for all age groups. This is true between the sexes in all countries as well as between countries. For all countries there was a general decline in the health status of the stock sample as individuals aged during the study period. This deterioration in health was accompanied by the occurrence of acute health shocks, though these did not increase in prevalence across the waves.

## 3. Analytical Methods

We estimate hazard functions for the transition to retirement. This is done using the stock-sampling approach, implemented by [35], which represents the transition to retirement as a discrete-time hazard specification, based on the [37] model. For this analysis the data are organised so that the unit of analysis is the time at risk of the event. In our case the event of interest is retirement and duration measures age of retirement. When in panel format the ECHP data has the necessary configuration. This organisation of the data and conditioning on the stock sampling—such that time periods prior to selection into the sample can be ignored—means that the estimation of a discrete-time hazard model is simplified, to the extent that any method suitable for the estimation of a binary responses may be used.

The theoretical construct of these models is summarised as follows, using notation based on [35]. The time at risk has a range from *t* = τ (wave 1) to *t* = τ + s_i_ where τ + s_i_ is the year when retirement occurs (complete duration data is indicated by δ_i_ = 1) or when the observation period for that individual ends (censored duration data, indicated by δ_i_ = 0). Thus each individual contributes s_i_ years of employment epoch data within the sample period. The probability of retiring at each *t* provides information on the distribution of retirement ages and the discrete-time hazard rate for retirement is defined as:

(5) $$h_{it}=\text{prob}[{\text{T}}_{i}=t|{\text{T}}_{i}\geq t;\;{\text{X}}_{\text{it}}]$$

where X*_it_* is a vector of covariates which may vary with time and T*_i_* is a discrete random variable representing the age at which the end of the epoch occurs.

The conditional probability of observing a retirement (event history) of someone with an incomplete employment epoch at a particular time period is:

(6) $$\text{prob}({\text{T}}_{i}>t|{\text{T}}_{i}>\tau -1)=\prod_{\text{t}=\tau }^{\tau +\text{si}}\;(1-h_{it})$$

and the conditional probability of observing a retirement (event history) of someone completing their employment at a particular time is:

(7) $$\text{prob}({\text{T}}_{i}=t|{\text{T}}_{i}>\tau -1)=h_{i\tau +si}\prod_{\text{t}=\tau }^{\tau +\text{si}}\;(1-h_{it})=(h_{i\tau +si}/\;(1-h_{i\tau +si}))\;\prod_{\text{t}=\tau }^{\tau +\text{si}}\;(-h_{it})$$

Accordingly the corresponding sample log-likelihood function of the observed retirement history data for the whole sample is:

(8) $$\text{log}\;\text{L}=\sum_{i=1}^{n}\delta_{\text{i}\;}\;\text{log}\;(h_{i\tau +si}/\;(1-h_{i\tau +si}))+\sum_{i=1}^{n}\sum_{t=\tau }^{\tau +\text{si}}\;\text{log}\;(1-h_{it})$$

Jenkins simplifies this log-likelihood by defining a binary outcome y_it_ = 1 if *t =* τ + s_i_ and δ_i_ = 1, y_it_ = 0 otherwise. Thus for those still working y_it_ = 0 for all periods, while for those who retire, y_it_ = 0, for all periods except the period in which they retire, when y_it_ = 1. The log-likelihood can then be expressed as:

(9) $$\text{log}\;\text{L}=\sum_{i=1}^{n}\sum_{t=\tau }^{\tau +si}\;{\text{y}}_{\text{it}\;}\;\text{log}\;(h_{i\tau +si}/\;(1-h_{i\tau +si}))+\sum_{i=1}^{n}\sum_{t=\tau }^{\tau +\text{si}}\;\text{log}\;(1-h_{it})$$

The specification is completed by specifying a complementary log-log hazard rate for *h_it_*:

(10) $$h_{it}=1-\text{exp}(-\text{exp}({\text{X}}_{it}\;\beta +\theta (t)))$$

where θ(*t*) is the baseline hazard and is modelled as a step function by using dummy variables to represent each period at risk (each year of age). This non-parametric form for the baseline hazard leads to a semi-parametric specification of the discrete-time duration model. Analyses are carried out using the discrete-time hazard model Stata program ‘pgmhaz8’ [38]. This program automatically incorporates frailty (unobserved heterogeneity) by assuming gamma distributed unobserved heterogeneity [39]. Dr. Nicoletti, *et al.* provide Monte Carlo evidence that these discrete hazard models are robust to misspecification of the form of the unobserved heterogeneity, whether parametric or nonparametric specifications are used, particularly for the estimated effects of the covariates [40].

Separate models were estimated using the estimated latent health stock variables, current and one-period lags, and the discrete acute health shock variables conditioned on the initial health stock. Each model includes the same demographic and financial variables. All models were estimated using the two alternative definitions of retirement: self-reported retirement and an expanded definition based on inactivity in the labour market.

## 4. Results

In the hazard models the discrete acute health shocks are conditioned on the initial value of the normalised latent health stock. The complete regression results are available from the authors on request. The key results for the same models in all countries are summarised in terms of hazard ratios in Table 4. The hazard ratio measures the proportional effect on the underlying (instantaneous) hazard of retiring of a one unit change in the value of the variable in question. Separate results are presented for men and women. The first column of results represents the models that includes the discrete acute health shock (≥1 std.dev.) while the second and third columns represent the model that includes current and lagged levels of the health stock.

Focusing on the first column of results, there is marked heterogeneity between countries in relation to the magnitude of response to health shocks. The countries where acute health shocks have the largest impact on the hazard of retirement are Ireland, Portugal, Greece and Spain for men and Ireland, Portugal, Denmark and Spain for women. For example, the hazard of early retirement is 4.5 times greater for Irish men who experience a health shock and 2.6 times greater for Irish women. One of these countries, Ireland, has an early retirement benefit due to reduced capacity to work as part of their public pension system [1]. Portugal has the highest ratio of disability-related pension expenditure to old age pension expenditure at 34% [1]. The remaining countries—Belgium, France, Italy and the UK—show predominantly smaller and non-statistically significant effects of acute health shocks with the exception of females in France.

Turning to the results in the second and third columns of Table 4, the countries showing a large effect of the discrete acute health shock also, as would be expected, show a similar effect of the current latent health stock variable when conditioned on the one-period lagged health stock. In this case the health stock is increasing in good health so hazard ratios less than 1 indicate that poorer health increases the hazard of retirement. The remaining countries who show either no or little effect of the discrete acute health shock tend to show some effect of the one-period lagged latent health stock suggesting that it is more chronic ill-health in those countries that influence the retirement decision.

There are differences between these EU countries in the incentives created by their social security and tax systems in relation to retirement, in the age at which early retirement is permitted and in the manner that pension benefits are accrued [5,6]. The decision to retire may be influenced by the standard and, in particular, early age of entitlement to public pension benefits. A second factor is the generosity of pension benefits. This is reflected in the replacement rate, defined as the ratio of post-retirement income to pre-retirement income, and the levels of pension wealth that people have at retirement. Finally the implicit marginal tax on continued work may influence the timing of retirement. The implicit marginal tax (or subsidy) on continued work reflects the change in net pension wealth from working an additional year, it compares the accrual of pension wealth during the year to the net wage earnings over the year [6,7]. Dr. Gruber, *et al.* define the ‘tax force to retire’ as the sum of the implied tax rates from age 55 through to age 69 [6].

**Figure 1.**
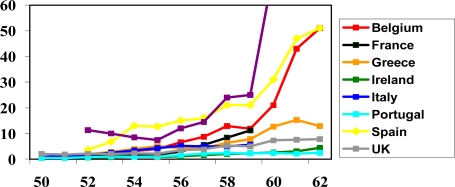
Retirement hazard ratio by year of age (3-year running average), by country, for males.

**Figure 2.**
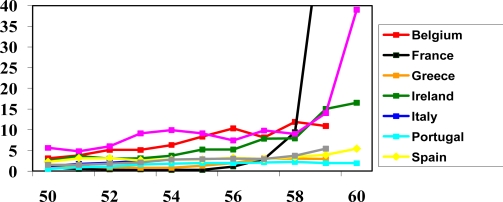
Retirement hazard ratio by year of age (3-year running average), by country, for females.

Not surprisingly then, the results reveal major differences between countries when we look at the age specific hazard ratios for retiring (Figures 1 and 2). For both sexes two of the four countries with the greatest effects of discrete acute health shocks (Portugal and Greece) show the least increase in the hazard of retiring with increasing age. These findings are robust across all models whether the health variables were included or excluded from the model.

For the countries used in our analysis [6] find the highest tax force, and hence the greatest financial incentive to retire early, in Italy followed by Belgium, with lower levels in France, then the UK and Spain. These countries, with the exception of Spain, show little effect of the discrete acute health shock but some effect of the lagged latent health stock suggesting that chronic ill-health may have more influence on the retirement decision. [7] presents the average implicit tax rate over the five years from age 60: this is less than the OECD average in Ireland, Portugal and the UK and above the average in Belgium, France and Spain. So the latter have the highest incentive to retire at 60 rather than later. This is supported by a pooled analysis of data combined across seven countries and including additional country-specific regressors representing the standard retirement age, average replacement rate and average implicit tax rate at 60 and 65 years (Table 5). Data on these variables were reported in [7].

The estimated effects for the other variables in the models are broadly stable across specifications that include the different health measures. There were however no systematic patterns across the different countries either for the education variables or the financial variables. The financial variables differed in some countries between the sexes, such that in Belgium higher personal income was associated with a decrease in the likelihood of retiring in females and an increase in males. However the important message in this paper is the consistency and stability of the effect of these other variables across the varying models within each country.

## 5. Tests of Robustness

We assessed the robustness of our specifications and models by (i) varying the stock samples used for both generating the health variables and running the models; (ii) using different lag periods for the latent health stock; and (iii) using an expanded definition of retirement.

We generated a set of latent health stock variables from each of the three stock samples. Thus seven discrete acute health shock variables were available for analysis: three generated from the initial age selected sample,as used in the models reported in our results section; two additional ones based on the normalised health stock generated using the stock sample of individuals employed or self-employed at wave 1; and two more based on the normalised health stock generated using the final stock sample. In addition, matching sets of current and lagged latent health stock variables were available from these stock samples. The derivation of the health variables had no substantive impact on their effect in all models nor did calculating the normalised health stock using all waves combined. Similarly adding additional lags in the latent health stock did not affect the substance of the results.

When the uncensored stock sample was used for analysis (discrete health shock hazard ratios are shown in Table 6) the results match those in Table 4 using the censored stock sample: Ireland, Portugal and Greece show the largest effects for men and Ireland, Portugal and Denmark show the largest effects for women. Further analysis showed that changing the age group selected in the initial stock sample, by extending the upper limit to 64 years of age and/or restricting the lower limit, still reveals the same countries showing large or little effect of the discrete acute health shocks on the retirement decision.

Comparing the measures of discrete acute health shock based on ≥1 sd decrement in normalised latent health stock or ≥ one category decrement in the adjusted or unadjusted SAH reveals the same message. The largest of these discrete health shocks, based on the number of adverse health events per shock, is associated in the majority of countries with a quantitatively greater effect on the hazard of retiring. There is no consistent pattern in the relative effects of the shocks based on the adjusted and unadjusted SAH (Table 6). These results do not imply a consistent direction of reporting bias that would lead to over-estimates of the impact of health on retirement.

Using the broader definition of retirement as labour market inactivity reveals that there is relative stability in the size of effect no matter which health measure is used and this is true in all countries and for both sexes (see Table 6).

## 6. Discussion

The measures of health used in past studies of retirement have ranged from subjective self-assessed health to detailed batteries of more objective questions about limitations, impairments or diseases. All are used as some approximation of the ‘true’ health state of the individual. Not surprisingly, given the range of measures used, the findings have shown variability in magnitude of effect though have been more consistent in showing that deterioration in health state is associated with an increased probability of retirement. The more recent literature has attempted to overcome the potential problems of reverse causality and measurement error in the subjective self-assessed measures by ‘purging’ this measure by regression on a set of health indicators, though these themselves are usually self-reported, and by using the predicted values from these regressions as a measure of a latent health stock [17,21–24]. Additionally by lagging this measure it is hoped to remove the risk of justification bias in relation to the retirement decision [10] and, when conditioned on initial values, gives a measure of an acute health shock.

The ECHP has a limited range of health indicators but the available set was used, in association with demographic variables, to instrument the self-assessed health measure recorded in the ECHP and to construct a latent health stock. This is then normalised separately for each country, in the manner of [23]. The latent health stock variable, with higher values corresponding to better health, was then used as an initial value to assess the effects of health stocks on the retirement decision. Three acute health shock variables were created by adapting the method of [33] using the normalised latent health stock variable, the objective SAH and the adjusted derived SAH. The largest of these acute shocks was associated with a greater magnitude of effect on the hazard of retiring than the more gradual declining latent health stock measure. All these measures of health reveal variation in effect across the nine countries and between men and women within some countries.

The effect, both in direction and magnitude, of other socioeconomic factors is robust across the different health measures and definitions of retirement. This is compatible with the work of [2] but contrary to that of [22]. It implies that the specific health measure used is unimportant when the focus is on the influence of the other factors. There are clear differences between these countries in their age-specific risk of retiring, reflecting the differences in their standard retirement age, age of early retirement and their social security systems in relation to an individual’s retirement decision. Overall, our results provide comparisons of the effects of health shocks across a range of European countries based on internationally comparable panel data from the ECHP. They provide evidence of the relationship of health to retirement in countries encompassing a wide spectrum of public pension systems. There is stability in the estimated effects of the different health measures across the two definitions of retirement and in the effects of the other factors including financial factors and education. Health shocks have a smaller impact on the hazard of retirement in the countries that have the strongest financial incentive to retire early.

## Figures and Tables

**Table 1.** Ordered probit coefficients for health variables: first wave.

	**Severly hampered**	**Moderately hampered**	**Illness or injury**	**Mental health problem**	**Inpatient stay**

Belgium					
Male	−2.099^4^	−1.165^4^	−0.702^4^	−0.861^3^	−0.170
Female	−2.247^4^	−1.182^4^	−0.616^4^	−0.856^4^	−0.345^1^

Denmark					
Male	−2.087^4^	−1.345^4^	−0.662^4^	0.172	−0.438^2^
Female	−2.216^4^	−1.469^4^	−0.763^4^	−0.460^1^	−0.388^2^

France					
Male	−1.850^4^	−0.955^4^	−0.370^1^	−1.098^1^	−0.646^4^
Female	−1.776^4^	−1.272^4^	−0.791^4^	−1.088^4^	−0.464^4^

Greece					
Male	−2.247^4^	−1. 500^4^	−0.327^1^	−0.790^3^	−0.636^4^
Female	−2.325^4^	−1.445^4^	−0.457^4^	0.428^1^	−0.497^4^4

Ireland					
Male	−2.374^4^	−1.378^4^	−0.719^4^	−0.911^4^	−0.497^4^
Female	−2.244^4^	−1.282^4^	−0.592^4^	−0.592^3^	−0.404^4^

Italy					
Male	−2.630^4^	−1.413^4^	−0.256	−0.407	−0.420^4^
Female	−2.521^4^	−1.230^4^	−0.614^4^	0.152	−0.559^4^

Portugal					
Male	−2.041^4^	− 1.072^4^	−0.680^4^	0.295	−0.835^4^
Female	−2.265^4^	−1.262^4^	−0.253^1^	0.016	−0.574^4^

Spain					
Male	−2.315^4^	−1.563^4^	−0.432^4^	−0.742^4^	−0.284^3^
Female	−2.349^4^	−1.518^4^	−0.610^4^	−0.601^4^	−0.241^2^

UK					
Male	−1.577^4^	−−−−−	−0.750^4^	−−−−−	−0.797^4^
Female	−1.380^4^	−−−−−	−0.577^4^	−−−−−	−0.776^4^

**Note:** The data for the UK sample does not distinguish levels of severity for ‘hamp’.

^1^ p < 0.05;

^2^ p < 0.01;

3 p < 0.005;

4 p < 0.001.

**Table 2.** Number of adverse health events per health shock^1^.

**Health shock**	**normalised health stock ≥ 1 sd decrement**		**Adjusted SAH ≥ 1 category decrement**		**Unadjusted SAH ≥ 1 category decrement**	
	mean	median (iqr)	mean	median (iqr)	mean	median (iqr)
Belgium	1.81	2 (1,2)	0.45	0 (0,1)	0.56	0 (0,1)
Denmark	1.78	1 (1,2)	1.03	0 (0,1)	0.66	0 (0,1)
France	2.20	2 (1,3)	2.10	2 (1,3)	0.97	0 (0,2)
Greece	1.88	2 (1,3)	1.31	1 (0,2)	0.61	0 (0,1)
Ireland	1.78	1 (1,2)	0.92	1 (0,1)	0.53	0 (0,1)
Italy	1.83	2 (1,2)	1.17	1 (1,2)	0.41	0 (0,1)
Portugal	2.07	2 (1,3)	1.08	1 (0,2)	0.85	0 (0,1)
Spain	1.89	2 (1,2)	1.77	2 (1,2)	0.56	0 (0,1)
UK	1.95	2 (1,3)	1.70	2 (1,3)	0.61	0 (0,1)
**overall mean**	1.91		1.28		0.64	
**coefficient of variation**	0.94%		17.5%		4.0%	

iqr: inter-quartile range;

^1^ sum of binary dummy variables illness, mental problem, inpatient, some limitation and 2* severe limitation; range 0–5. In all countries those individuals who did not have an acute health shock had significantly less adverse health events than those that did.

**Table 3.** Stock samples recruitment by country

		**45–59 yrs**	**Censored sample to remove individuals over state retirement age**									
			**Employed wave 1 & Censored^1^**	**Present wave 8**	**Sample analysed**	**%^2^**	**Self report retirement**	**%^3^**	**Expanded retirement**	**%^3^**	**Discrete acute health shocks**	**Range of shocks by wave (%)**
Belgium	m	649	513	173	445	69	85	19	106	24	117	3–8
	f	673	265	81	265	39	61	23	77	29	59	4–11
Denmark	m	686	571	296	511	74	77	15	105	21	145	5–7
	f	715	498	265	443	62	103	23	138	31	181	6–10
France	m	1,410	1,115	428	964	68	171	18	235	24	430	7–12
	f	1,443	794	313	687	48	139	20	168	24	306	7–13
Greece	m	1,433	1,183	604	1,091	76	236	22	304	28	335	5–8
	f	1,473	542	186	482	33	191	40	207	43	106	4–9
Ireland	m	1,093	870	265	711	65	92	13	128	18	195	4–9
	f	1,174	318	84	247	21	77	31	85	34	79	6–14
Italy	m	2,146	1,677	532	1,483	69	373	25	424	29	294	3–6
	f	2,201	660	143	559	25	120	21	131	23	101	3–6
Portugal	m	1,178	945	561	894	76	189	21	238	27	369	5–11
	f	1,356	625	321	594	44	191	32	214	36	215	5–10
Spain	m	1,838	1,355	542	1,113	61	225	20	327	29	309	5–8
	f	1,971	513	201	429	22	151	35	171	40	141	7–10
UK	m	913	699	479	657	72	164	25	200	30	246	5–11
	f	1,053	635	354	578	55	183	32	206	36	255	6–14
total		23,405	13,766	5,828	12,153	52	2,828	23	3,464	29		

^1^ Sample right censored to remove individuals over state retirement age;

^2^ as percent of eligible age group;

^3^ as percent of those analysed.

**Table 4.** Hazard ratios for the health variables.

	**Males**			**Females**		
	**Discrete Acute health shock**	**Lagged latent health stock (1 period)**	**Current latent health stock**	**Acute health shock**	**Lagged latent health stock (1 period)**	**Current latent health stock**
**Ireland**	4.5^4^	0.84	0.46^4^	2.6^1^	0.83	0.53^3^
	(2.7, 7.7)	(0.66, 1.1)	(0.38, 0.56)	(1.1, 6.4)	(0.54, 1.3)	(0.37, 0.76)
**Portugal**	3.7^4^	0.89	0.42^4^	3.7^4^	1.0	0.52^4^
	(2.5, 5.5)	(0.71, 1.1)	(0.35, 0.52)	(2.5, 5.5)	(0.80, 1.3)	(0.41, 0.66)
**Greece**	3.5^4^	1.1	0.55^4^	1.8	0.91	0.77
	(2.3, 5.2)	(0.89, 1.4)	(0.47, 0.64)	(0.89, 3.7)	(0.66, 1.3)	(0.58, 1.0)
**Spain**	1.8^1^	0.52^4^	0.60^4^	2.0^*^	0.77^1^	0.80
	(1.0, 3.1)	(0.41, 0.66)	(0.49, 0.72)	(1.2, 3.3)	(0.60, 0.98)	(0.63, 1.0)
**Denmark**	2.1	0.44^2^	0.59	2.6^1^	0.58^1^	0.43^4^
	(0.96, 4.8)	(0.24, 0.80)	(0.34, 1.0)	(1.2, 5.7)	(0.35, 0.95)	(0.27, 0.68)
**Italy**	1.3	0.83^1^	0.89	0.96	1.1	0.97
	(0.79, 2.0)	(0.72, 0.96)	(0.77, 1.0)	(0.39, 2.4)	(0.83, 1.5)	(0.72, 1.3)
**France**	1.2	0.67^3^	0.85	1.5	0.59^1^	0.61^1^
	(0.74, 2.0)	(0.54, 0.85)	(0.69, 1.1)	(0.61, 3.8)	(0.38, 0.90)	(0.41, 0.90)
**UK**	1.1	0.71^1^	0.89	1.5	0.78^1^	0.82
	(0.59, 2.0)	(0.52, 0.97)	(0.67, 1.2)	(0.92, 2.6)	(0.62, 0.99)	(0.66, 1.0)
**Belgium**	0.92	0.70^1^	0.87	1.0	0.92^1^	1.0
	(0.32, 2.6)	(0.51, 0.95)	(0.62, 1.2)	(0.30, 3.4)	(0.43, 0.90)	(0.66, 1.6)

**(95% CI)**

^1^ p < 0.05;

^2^ p < 0.01;

^3^ p < 0.005;

^4^ p < 0.001

**Table 5.** Combined analysis of effects of retirement age, pension and tax systems on retirement decision in seven European Union countries (Belgium, France, Ireland, Italy, Portugal, Spain & UK).

	**Health variables**		**Retirement ages**	**Replacement rate**	**Implicit tax rates**	
	**Initial stock**	**Acute shock**	**Standard**	**Age 60**	**Age 60**	**Age 65**
**Self reported Retirement**						
**Males**	−0.16382	0.52212	−0.38784	−0.12542	0.12597	0.07955
**hr**	0.85 **4**	1.7 **4**	0.68**4**	0.88 **4**	1.1 **1**	1.1 **3**
	**No health variables in model**		−0.4160	−0.14138	0.16037	0.06885
			0.66 **4**	0.87 **4**	1.2 **2**	1.1 **2**
**Females**	−0.18731	0.45594	−0.07986	−0.13286	0.08524	0.06698
**hr**	0.83**4**	1.6**4**	0.92	0.88**4**	1.1	1.1**1**
	**No health variables in model**		−0.07890	−0.14487	0.11912	0.06714
			0.92	0.87**4**	1.1**1**	1.1**1**

Standard retirement age analysed as number of years that country’s value was above Italy which had lowest age in this group of countries. The variable is grouped in five year intervals: 1–5 yrs = 1; 6–10 yrs = 2 etc. The replacement rate and implicit tax rate are analysed as ordered bands of width 20% i.e., 0–19% = 1; 20–39 = 2 etc.

^1^ p < 0.05;

^2^ p < 0.01;

^3^ p < 0.005;

^4^ p < 0.001.

**Table 6.** Hazard ratios—discrete acute health shocks, stock sample 2 used.

	**Self report**			**Expanded retirement**		

	**Discrete acute health shock**			**Discrete acute health shock**		

	**Normalised health stock ≥1 sd decrement**	**Adjusted SAH ≥ 1 category decrement**	**Unadjusted SAH ≥ 1 category decrement**	**Normalised health stock ≥1 sd decrement**	**Adjusted SAH ≥ 1 category decrement**	**Unadjusted SAH ≥ 1 category decrement**

Ireland						
Male	5.00**3**	3.21**3**	1.51	3.35**3**	2.12**2**	1.53**1**
Female	1.91	1.10	1.65	2.06	1.30	1.35

Portugal						

Male	3.34**3**	1.91**2**	1.94**2**	3.35**3**	1.77**2**	1.92**3**
Female	2.72**3**	1.76**2**	1.77**2**	2.85**3**	1.99**3**	1.88**3**

Greece						

Male	3.13**3**	1.75**2**	2.01**3**	2.66**3**	1.69**2**	1.95**3**
Female	1.67	1.72**1**	1.69**2**	1.96**1**	2.05**2**	1.81**2**

Spain						

Male	1.94**2**	1.84**2**	1.41**1**	1.68**2**	1.62**1**	1.21
Female	1.62	1.55	1.38	1.99**2**	1.55	1.24

Denmark						

Male	0.94	0.44	0.61	1.26	0.94	0.75
Female	2.05**1**	1.44	1.75**1**	1.28	1.41	1.22

Italy						

Male	1.47**1**	1.29	1.44**2**	1.51**1**	1.32	1.43**2**
Female	0.72	0.92	1.33	0.70	0.97	1.32

France						

Male	0.79	1.17	0.96	0.81	1.06	0.93
Female	1.84**2**	1.75**2**	1.18	1.78**2**	1.72**2**	1.02

UK						

Male	1.38	1.06	0.83	1.43	1.32	0.83
Female	1.53**1**	1.29	1.07	1.53**1**	1.27	1.15

Belgium						

Male	1.04	.96	1.15	1.16	1.19	1.07
Female	1.74	2.03	0.60	1.76	1.81	0.68

^1^ p < 0.05;

^2^ p < 0.01;

^3^ p < 0.001.
